# GATA4 Directly Regulates *Runx2* Expression and Osteoblast Differentiation

**DOI:** 10.1002/jbm4.10027

**Published:** 2018-01-03

**Authors:** Aysha B Khalid, Alexandria V Slayden, Jerusha Kumpati, Chanel D Perry, Maria Angeles Lillo Osuna, Samantha R Arroyo, Gustavo A Miranda‐Carboni, Susan A Krum

**Affiliations:** ^1^ Department of Orthopaedic Surgery and Biomedical Engineering University of Tennessee Health Science Center Memphis TN USA; ^2^ Department of Medicine University of Tennessee Health Science Center Memphis TN USA; ^3^ Center for Cancer Research University of Tennessee Health Science Center Memphis TN USA

**Keywords:** GATA4, BONE, OSTEOBLASTS, RUNX2

## Abstract

GATA4 is a zinc‐finger transcription factor that is a pioneer factor in various tissues and regulates tissue‐specific gene regulation. In vivo deletion of *Gata4* using Cre‐recombinase under the control of the *Col1a1* 2.3 kb promoter showed significantly reduced values for trabecular bone properties by microCT analysis of femur and tibia of 14‐week‐old male and female mice, suggesting GATA4 is necessary for maintaining normal adult bone phenotype. Quantitative PCR analysis revealed higher expression of *Gata4* in trabecular bone compared with cortical bone, suggesting a role for GATA4 in maintaining normal trabecular bone mass. In vivo and in vitro, reduction of *Gata4* correlates with reduced *Runx2* gene expression, along with reduced osteoblast mineralization. To determine if *Runx2* is a direct target of GATA4, chromatin immunoprecipitation (ChIP) was performed, and it demonstrated that GATA4 is recruited to the two *Runx2* promoters and an enhancer region. Furthermore, when *Gata4* is knocked down, the chromatin at the *Runx2* region is not open, as detected by DNase assays and ChIP with antibodies to the open chromatin marks H3K4me2 (histone 3 lysine 4 dimethylation) and H3K27ac (histone 3 lysine 27 acetylation) and the closed chromatin mark H3K27me2 (histone 3 lysine 27 trimethylation). Together, the data suggest that GATA4 binds near the *Runx2* promoter and enhancer and helps maintain open chromatin to regulate *Runx2* expression leading to bone mineralization. © 2017 The Authors. *JBMR Plus* is published by Wiley Periodicals, Inc. on behalf of the American Society for Bone and Mineral Research.

## Introduction

Osteoblast differentiation is controlled by directly inducing the expression of, or regulating the activities of, the “master” osteoblast transcription factor RUNX2. *Runx2* is expressed in undifferentiated mesenchymal cells, hypertrophic chondrocytes, and early in osteoblast differentiation, where it turns on other differentiation and mineralization genes, including alkaline phosphatase and osteocalcin (*Bglap*).^1^ The *Runx2* promoter is controlled epigenetically, as increased levels of histone 3 lysine 4 methylation (H3K4me) and histone 3 lysine 27 acetylation (H3K27ac) correlate with osteoblast‐specific expression.^2^ However, the transcription factors that regulate *Runx2* promoter epigenetics and subsequent mRNA expression early in osteoblastogenesis are not well understood.

GATA4 is a zinc‐finger transcription factor that binds to the consensus region “GATA.” It is important for the development of the heart and intestines early in embryogenesis, as it mediates tissue specificity. *Gata4* knockout mice die early in embryogenesis due to the heart and intestinal defects.^3^, ^4^ We have recently demonstrated that GATA4 is also a critical transcription factor for osteoblast differentiation, and GATA4 controls osteoblast function by regulating the balance between TGFβ and BMP pathways.^5^ Conditional *Gata4* knockout mice (cKO) with Cre expression driven by the 2.3‐kb promoter of *Col1a1* showed reduced trabecular bone mass and exhibited both appendicular and cranial defects in newborn mice. GATA4 has also been shown to act as a regulator of osteoblasts by controlling estrogen receptor recruitment to the chromatin in a tissue‐specific manner.^5^, ^6^

Given the early role of GATA4 in regulating bone mass in newborn cKO mice and the co‐localization of GATA4 and RUNX2 in trabecular bone and in calvaria,^6^ we examined if GATA4 regulates osteoblast differentiation via *Runx2* in vitro and in vivo. We present evidence that GATA4 maintains open chromatin at both the *Runx2* promoters and an enhancer to upregulate *Runx2*, the master regulator of osteoblast differentiation, and to control proper adult bone formation in vivo. We suggest that GATA4 is critical for the epigenetic mechanisms of tissue‐specific differentiation of osteoblasts.

## Materials and Methods

### Animals

Animal experiments were approved by the Institutional Animal Care and Use Committee at the University of Tennessee Health Science Center. Animals were maintained in a specific pathogen‐free environment at 20°C to 26°C with a relative humidity of 30% to 70% and a 12‐hour light/dark cycle. Commercial rodent chow (LM‐485, Teklad, Madison, WI, USA) and drinking water were available *ad libitum*. GATA4^fl/fl^ mice were purchased from The Jackson Laboratory (Bar Harbor, ME, USA), where they were generated by homologous recombination in which the region containing the GATA4 coding sequence upstream of exon 3 and downstream of exon 5 was replaced with a loxP‐flanked thymidine kinase/neomycin phosphotransferase expression (tk/neo).^7^ The homozygous GATA4^fl/fl^ mice was backcrossed for 10 generations to FVB background.^(5)^ Mice that were heterozygous for the deletion were crossed to collagen A1(Col1A1) (2.3 kb)‐Cre mice (FVB‐Tg(Col1a1‐cre)1Kry/Mmucd) (Mutant Mouse Regional Resource Centers [MMRRC]) to produce homozygous GATA4 cKO and wild‐type littermate control mice. Fourteen‐week‐old adult (12 wild type [WT] and 12 knockouts [cKO]) were euthanized and the right hindlimbs cleaned and stored in phosphate‐buffered saline (PBS) at −20°C for micro‐computed tomography (μCT) until measurements were made. The left femur was transferred to formalin and the left tibia was transferred to ethanol and stored at 4°C.

### Micro‐computed tomography

Tibias, femora, and vertebrae from 14‐week‐old mice were stored in PBS and then scanned using a Scanco μCT 40 (Brüttisellen, Switzerland) set at 55 kVp/109 µA. The entire femur and tibia were scanned in the sample holder with 12.3‐mm diameter at medium resolution. These tubes were filled with PBS and the top of the tube was covered with Parafilm (American National Can, Chicago, IL, USA) to prevent dehydration. A scout view of each bone was taken and the sample height was adjusted to ensure the bone was within the field of view. The tibias and femora images were obtained at 6‐μm resolution. The integration time and Gaussian filter used for these samples was 300 ms and 1, respectively. Solid three‐dimensional models were reconstructed from these images automatically after completion of each cone‐beam image stack with the built‐in software. The trabecular parameters were calculated on 200 slices of trabecular bone from a region just below the growth plate as described in Khalid and colleagues^8^ with a threshold of 250 and quoted using American Society of Bone and Mineral Research nomenclature. μCT was also used to measure the cortical properties of each diaphysis on 100 slices of cortical bone from region closest to the center of the shaft of the bone. The L_5_ vertebrae were isolated using the μCT X‐ray scout view and scanned with an 8‐µm voxel size. A section of the vertebral body measuring 0.5 mm immediately cranial to the caudal end plate was selected for analysis.^9^

### Bone histology

Bone histology was performed at the Histomorphometry and Molecular Analyses Core (HMAC) at the University of Alabama at Birmingham School of Medicine.

### Immunohistochemistry

Formalin‐fixed paraffin‐embedded samples were processed through standard deparaffinization protocols. Antigen retrieval was performed in 90°C citrate buffer until the temperature reached 55°C. The tissue was then incubated in blocking buffer (5% normal goat serum, 2.5% bovine serum albumin [BSA] in PBS at pH 7.5) for 30 minutes. Anti‐RUNX2 (clone M‐70, Santa Cruz Biotechnology, Dallas, TX, USA) antibodies were incubated overnight at 4°C in a humidified chamber followed by the DAKO Envision Visualization system and counterstaining with hematoxylin.

### Isolation and culture of osteoblasts

Mouse calvarial osteoblasts were isolated from 2‐day‐old CD1 mice by sequential collagenase digestion, as previously described.^10^ In brief, the cells were incubated for 40 minutes in αMEM‐1.0 mg/mL collagenase P‐1.25% trypsin at 37°C. The cells were then washed in α‐MEM and then incubated in αMEM‐1.0 mg/mL collagenase P‐1.25% trypsin for 1 hour at 37°C. Collagenase digestion was stopped by addition of complete α‐MEM media containing 10% FBS. The cells from second digest were seeded in a 6‐well plate at a density of 2 × 10^5^ cells/well and allowed to adhere overnight. To determine the effect of knocking out GATA4 on mineralization, the silenced shC and shGATA4 mouse calvarial cells (lentivirus silencing as described below) were differentiated in mineralization medium supplemented with 50 μg/mL L‐ascorbic acid 2‐phosphate and 5 mM β glycerophosphate. Cells were maintained under these conditions for 14 days with media replaced every 3 days. Experiments were performed in α‐MEM media with 10% fetal bovine serum (Omega Scientific, Tarzana, CA, USA). Differentiation was confirmed by gene expression and fixing the cells and staining with Alizarin Red.

Mouse bone marrow cells were isolated from long bones of 3‐month‐old WT and GATA4 KO mice. Each hindlimb was cleaned off by scraping the diaphysis and pulling the tissue toward the end of the bones. The ends of the femur and tibia just below the end of marrow cavity were chopped with sharp rongeur. A 27‐gauge needle attached to a 10‐mL syringe containing basal media was then used to flush out the bone marrow. The bone marrow cells were then seeded in a 6‐well plate at a seeding density of 2 × 10^6^ cells/well and left undisturbed for 5 days in MesenCult MSC Basal Medium (Mouse, StemCell Technologies, Vancouver, Canada). To determine the effect of knocking out *Gata4* on mineralization, the bone marrow cells were treated for 14 days with α‐MEM media supplemented with 50 μg/mL L‐ascorbic acid 2‐phosphate and 5 mM β glycerophosphate with media changed every 3 days. After 14 days, the difference between WT and cKO mineralization was confirmed by fixing the cells and staining with Alizarin Red.

### Isolation of trabecular and cortical bone

Wild‐type femurs were prepared by removing muscle and flushing the bone marrow. The epiphysis was cut from the diaphysis for the trabecular and cortical bone, respectively.

### Cells

U2OS cells were purchased from American Type Culture Collection (Manassas, VA, USA) and validated yearly by Genetica DNA Laboratories. The cells were grown in DMEM‐F12 with 10% fetal bovine serum. The cells were maintained at 37°C with 5% CO_2_.

### Knockdown of *Gata4*

Lentivirus shC (a short hairpin that does not recognize any mammalian DNA) and shGATA4 were purchased from Sigma‐Aldrich (St. Louis, MO, USA). Two different shRNA from The RNAi Consortium (TRC) in pLKO vector were used to knock down mouse *Gata4* (TRCN0000095215: CCGGCCCAATCTCGATATGTTTGATCTCGAGATCAAACATATCGAGATTGGGTTTTTG and TRCN0000095217: CCGGCATCTCCTGTCACTCAGACATCTCGAGATGTCTGAGTGACAGGAGATGTTTTTG). The calvarial cells were plated in six‐well plates at a seeding density of 2 × 10^5^ cells per well. After 24 hours, the cells were infected with lentivirus in α‐MEM with 8 µg/mL polybrene per well. The plates were centrifuged at 1400*g* at 30°C for 45 minutes and left undisturbed for 24 hours after which the cells were washed with PBS and mineralization media was added. The mineralization assay was continued for 14 days with media replacement every 3 days. The knockdown of Gata4 was confirmed by qPCR.

Knockdown of GATA4 in U2OS cells was performed as described.^6^

### RNA and qPCR

Total RNA was isolated from mouse cells differentiated in osteogenic media for 14 days using TRIzol (Invitrogen, Carlsbad, CA, USA). cDNA was prepared using Superscript III First Strand Synthesis Kit according to the manufacturer's guideline and then quantified using TaqMan Universal Master Mix II (Applied Biosystems, Foster City, CA, USA) or SYBR Green (ThermoFisher Scientific, Waltham, MA, USA). The qPCR cycling conditions for TaqMan were initial denaturation at 50°C for 2 minutes; 95°C for 10 minutes, followed by 40 cycles of 95°C for 15 seconds and 60°C for 1 minute. The SYBR Green conditions used were 95°C for 10 minutes, followed by 40 cycles of 95°C for 15 seconds and 60°C for 1 minute. The oligonucleotide‐specific primers and probes used are listed in Supplemental Table S2. For analysis of the data, the values were normalized to β‐actin values.

### Protein extraction and Immunoblotting

Immunoblotting was performed on the osteoblasts from shC and shGATA4 samples. The cells were lysed on ice in EBC lysis buffer (50 mM Tris [pH 8], 120 mM NaCl, 0.5% Nonidet P‐40) supplemented with a protease inhibitor cocktail (Complete; Roche Applied Science, Indianapolis, IN, USA). Protein concentrations were quantified using the Bradford method. Proteins samples were boiled for 10 minutes at 95°C in sodium dodecyl sulfate (SDS) loading buffer and separated using SDS‐PAGE and then transferred on a polyvinylidene difluoride membrane (GE Healthcare Bio‐Sciences, Pittsburgh, PA, USA) via transfer apparatus according to the manufacturer's protocols (Bio‐Rad Laboratories, Hercules, CA, USA). The membrane was then probed with Runx2 (1:100) (Santa Cruz Biotechnology) and β‐actin (1:5000) (Cell Signaling, Danvers, MA, USA) after incubating with 5% milk in TBST overnight at 4°C. The proteins were detected by using 1:10,000 dilution of horseradish peroxidase‐conjugated secondary antibodies (anti‐rabbit or anti‐mouse) along with peroxidase substrate for chemiluminescent detection (ThermoFisher Scientific). Membranes were exposed to X‐ray film and developed with ECL system (ThermoFisher Scientific). Each immunoblot was performed in triplicate and the protein levels were normalized to β‐actin for individual samples.

### Alizarin Red assay

Osteoblasts were differentiated for 14 days and then fixed in 50% ethanol for 15 minutes at 4°C, followed by a 30‐minute incubation with 1% Alizarin Red S (wt/vol with 0.1% ammonium hydroxide). The stain was then washed with distilled water, dried, and photographed. The amount of mineral content was measured by eluting the Alizarin Red stain with 10% cetylpyridinium chloride and the optical density was measured at OD 570 nm.

### Chromatin immunoprecipitation

For chromatin immunoprecipitation, the calvarial cells were plated at a seeding density of 2 × 10^5^ cells and left undisturbed for 24 hours before silencing using lentivirus directed toward shC or shGATA4 as mentioned above. After 24 hours of silencing, the cells were washed with PBS and complete α‐MEM media was added to each well and left for and additional 3 days after which ChIP was performed using truChIP Ultra Low Cell Chromatin Shearing Kit (Covaris, Inc., Woburn, MA, USA) (see supplemental information). qPCR using SYBR Green Mastermix (Life Technologies, Carlsbad, CA, USA) was used to amplify the immunoprecipitated DNA.

GATA4‐Flag*‐*biotin mice (Gt(ROSA)26Sortm1(birA)Mejr Gata4tm3.1Wtp/J)^11^ were obtained from The Jackson Laboratory. The calvarial cells from 2‐day‐old GATA4‐Flag*‐*biotin pups were isolated as described above for wild‐type calvaria and grown under appropriate cell culture conditions in α‐MEM media with 10% fetal bovine serum in 15‐cm dishes. Once confluent, the cells were fixed with 37% formaldehyde for 10 minutes, and the excess formaldehyde was quenched with glycine for an additional 5 minutes as per the Magna ChIP A/G chromatin immunoprecipitation kit protocol (EMD Millipore, Billerica, MA, USA). Wild‐type CD1 mice were used as a control under identical conditions. The chromatin was lysed according to Millipore protocol and sonicated using a Biorupter sonicator (Diagenode, Denville, NJ, USA) and incubated for 1 hour at 4 °C with protein A beads to preclear the sheared chromatin followed by overnight incubation at 4 °C with streptavidin beads (ThermoFisher Scientific). After the incubation, the samples were sequentially washed with cold SDS wash buffer (2% SDS), high‐salt buffer (50 mM HEPES pH 7.5, 500 mM NaCl, 1 mM disodium EDTA, 0.1% (w/v) sodium deoxycholate, 1% (v/v) Triton X‐100), LiCl buffer (10 mM Tris‐Cl pH 8.1, 250 mM LiCl, 1 mM disodium EDTA, 0.5% (v/v) Nonidet P‐40 (NP‐40), 0.5% (w/v) sodium deoxycholate), TE buffer (10 mM Tris‐Cl, pH 7.5, 1 mM disodium EDTA), and Low Stringency IP Wash Buffer (EMD Millipore).^12^ To reverse the cross‐links, the IP beads and the input were resuspended in Millipore elution buffer and placed on a heat block for 2 hours at 65 °C and an additional 15 minutes at 95 °C, after which the DNA was validated by qPCR. Each ChIP was performed in biological triplicates. qPCR was performed as described above with primers listed in Supplemental Table S2. An intergenic region on chromosome 2 that tested negative in the ChIPs for the included histone marks and GATA4 was used for normalization (mouse negative [mo. −ve] primers).

### DNase

Chromatin with an equivalent of 2 μg of DNA was treated with variable amounts of DNase I (0, 2, 4 Units) in 200 μL total in 1× RSB (10 mM Tris pH 7.4, 10 mM NaCl, 3 mM MgCl2, 0.3% NP‐40) for 3 minutes at 37°C. DNA was then purified using DNeasy Tissue kit (Qiagen, Valencia, CA, USA) and qPCR was performed with primers in Supplemental Table S2.

### Luciferase

U2OS osteosarcoma cells were plated in 24‐well tissue culture dishes and transfected with the rat 0.6 kb Runx2‐promoter‐luciferase (a kind gift from Dr Gary S Stein),^13^ pcDNA3‐GATA4 (a kind gift from Dr Michael Parmacek^14^), and pRL‐SV40 (Promega, Madison, WI, USA) as a transfection control.

The Runx2‐promoter luciferase was mutated by PCR mutagenesis,^15^ using the following primers: gctccttcagcatttgtattctggccaaatcctcatgagtcacaaa and tttgtgactcatgaggatttggccagaatacaaatgctgaaggagc. The sequence “GATA” starting at position 359 was mutated to “GCCA.”

Each transfection was performed in triplicate and the entire assay was repeated at least 3 times. Twenty‐four hours after transfection, cells were assayed according to the manufacturer's protocol (Promega, Dual‐Luciferase Reporter Assay System). To obtain RLU (relative luciferase units), all values were normalized as follows: The raw firefly luciferase value was divided by the internal Renilla Luciferase value (pRL‐SV40) and then divided by the vector control.

### RNA in situ (RNAscope)

*Gata4* and *Runx2* mRNA were detected in situ by RNAscope technology (2.5 High Definition, Advanced Cell Diagnostics, Newark, CA, USA) in paraffin‐embedded wild‐type and cKO femurs according to the manufacturer's recommendations. Probes were designed by Advanced Cell Diagnostics.

### Statistical analysis

The data shown are the mean ± standard deviation. Statistical analyses were made using Sigma Plot (version 11.0, Systat Software Inc., San Jose, CA, USA). Data were analyzed using a two‐tailed Student's *t* test and a *p* value less than 0.05 was considered statistically significant. All experiments were performed with both biological and technical triplicates, except for mouse studies where *n* = 12.

## Results

### GATA4 regulates the bone mineral density of adult mice

Previously, we found that newborn cKO (*Gata4^fl/fl^*/Cre+) mice were not born at the expected Mendelian ratio, and μCT analysis of the newborn femur and vertebra showed reduced trabecular bone properties and skeletal defects.^5^ After an expansion of the breeding colony, 429 mice were since bred to obtain 7.9% cKO adult mice (25% was expected based on breeding *Gata4^fl/fl^* female mice with *Gata*4^fl/+^/Cre+ male mice). A statistically significant cohort of 14‐week‐old *Gata4* cKO mice was obtained to analyze the adult bone phenotype. To ensure that the surviving mice are still knockouts, RNA in situ using RNAscope technology was used to monitor *Gata4* gene expression in the trabecular bone of femurs from wild‐type and cKO mice. Indeed, there is less *Gata4* expression in the osteoblasts (Runx2‐positive cells) of cKO mice, as would be expected (Supplemental Fig. S1); however, the relative expression of GATA4 in different tissues and cells may vary.

**Figure 1:**
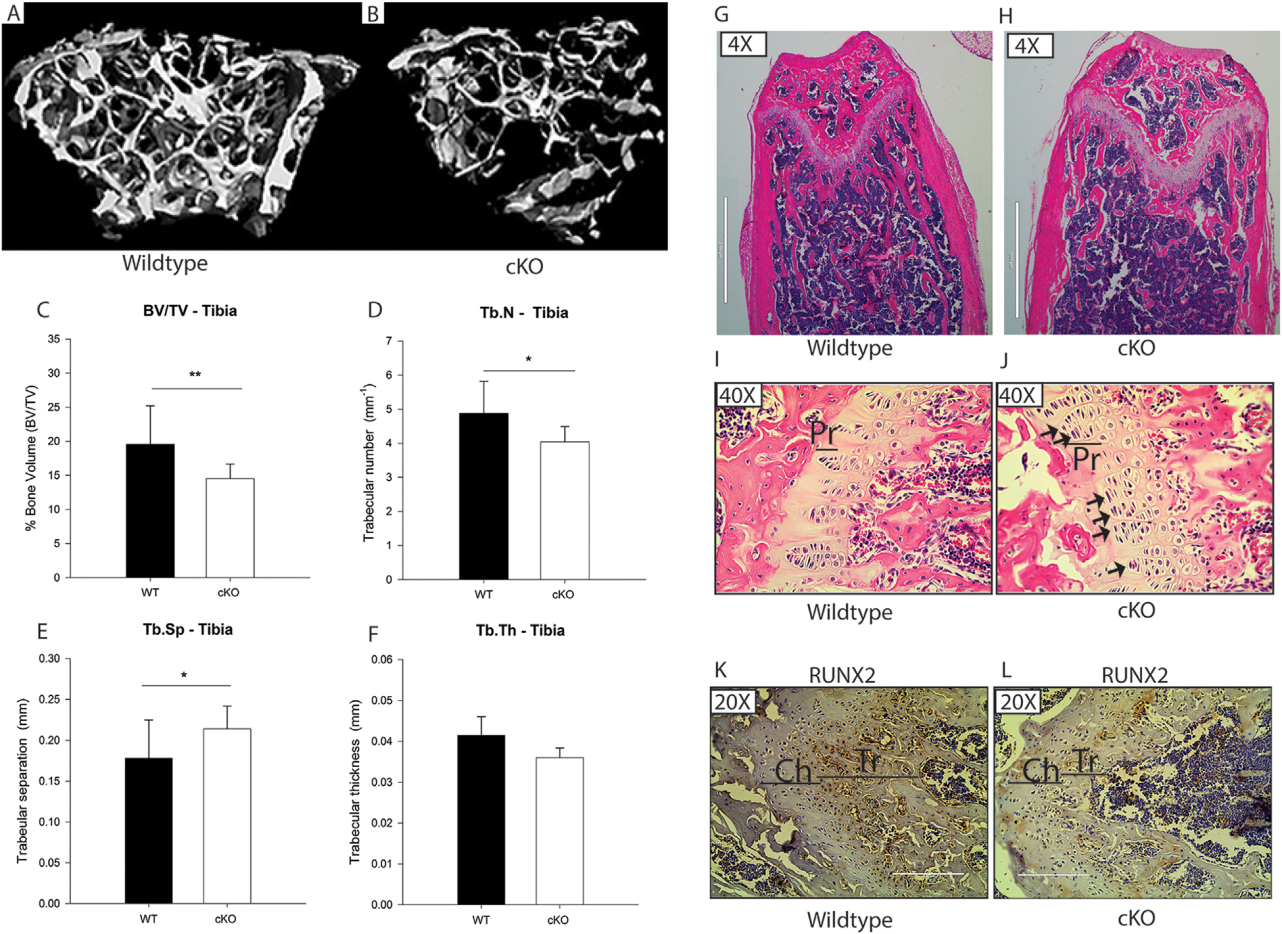
Trabecular bone volume is decreased in GATA4 cKO mice. (*A*, *B*) μCT reconstruction of trabecular bone in the distal tibia metaphysis of 14‐week‐old female mice. (*C–F*) Mean percentage bone volume (BV/TV), trabecular number (Tb.N), separation (Tb.Sp), and thickness (Tb.Th). Black bars indicate wild‐type (WT) mice; white bars indicate GATA4 conditional knockout (cKO) mice. Data are mean ± standard deviation from 12 WT and 12 cKO mice. Student's *t* test: *p < 0.05, **p < 0.01 compared with WT. (*G–J*) Section of femur stained with H&E and (*K*, *L*) RUNX2. Pr = proliferating chondrocytes. Arrows indicate binucleate chondrocytes. Ch = chondrocytes. Tr = trabecular bone. Scale bars = 1000 μm (*G*, *H*); 200 μm (*K*, *L*).

The percentage bone volume (bone volume [BV]/tissue volume [TV]) was significantly reduced in tibia of adult female cKO mice compared with WT littermates, together with a significant decrease in the trabecular number and a significant increase in trabecular separation, whereas no change was found in trabecular thickness (Fig. 1 *A–F*). No significant changes in cortical bone thickness nor porosity were detected between *Gata4* cKO adult mice and their littermate controls (Supplemental Fig. S2). The expression of *Gata4* mRNA was compared in trabecular and cortical bone, and *Gata4* is sixfold higher in trabecular bone (Supplemental Fig. S3), suggesting that GATA4 has a specific role in trabecular bone. Similar μCT results were obtained in male mice (Supplemental Fig. S4) and in the femurs of both male and female mice (Supplemental Table S1). The vertebrae of cKO also showed reduced BV/TV, decreased trabecular number, and increased trabecular separation (Supplemental Fig. S5).

Trabecular changes in the *Gata4* cKO were also confirmed by histological analyses of femurs from 14‐week‐old mice. Hematoxylin and eosin (H&E) staining of bone sections from *Gata4* cKO mice showed a reduced percentage bone volume and number of trabeculae, whereas the trabecular spacing was increased compared with wild‐type mice (Fig. 1 *G*, *H*). H&E staining also showed abnormalities in the cKO chondrocytes; the cKO proliferative zone was increased (Fig. 1 *I*, *J*) compared with the WT bone. In addition, there are more binucleate chondrocytes in the cKO bones.

Because the observed differences in the chondrocytes and trabecular bone at the growth plate mimic the expression pattern of RUNX2,^16^ we hypothesized that in the absence of GATA4, RUNX2 protein levels would be decreased in osteoblasts and chondrocytes in the growth plate. To address this hypothesis, RUNX2 immunohistochemistry was performed on sections from WT and cKO bones. RUNX2 was observed in the hypertrophic chondrocytes and osteoblasts in WT bones, as demonstrated previously.^(16)^ Significantly less RUNX2 staining was observed in the cKO growth plate and trabecular bone (Fig. 1 *K*, *L*). Furthermore, there is less *Runx2* mRNA in the femurs of cKO mice (Supplemental Fig. S1). These results suggest that *Runx2* is a target of GATA4 gene regulation at the growth plate, affecting both normal osteoblast and chondrocyte differentiations.

### GATA4 affects osteoblast mineralization in vitro

**Figure 2:**
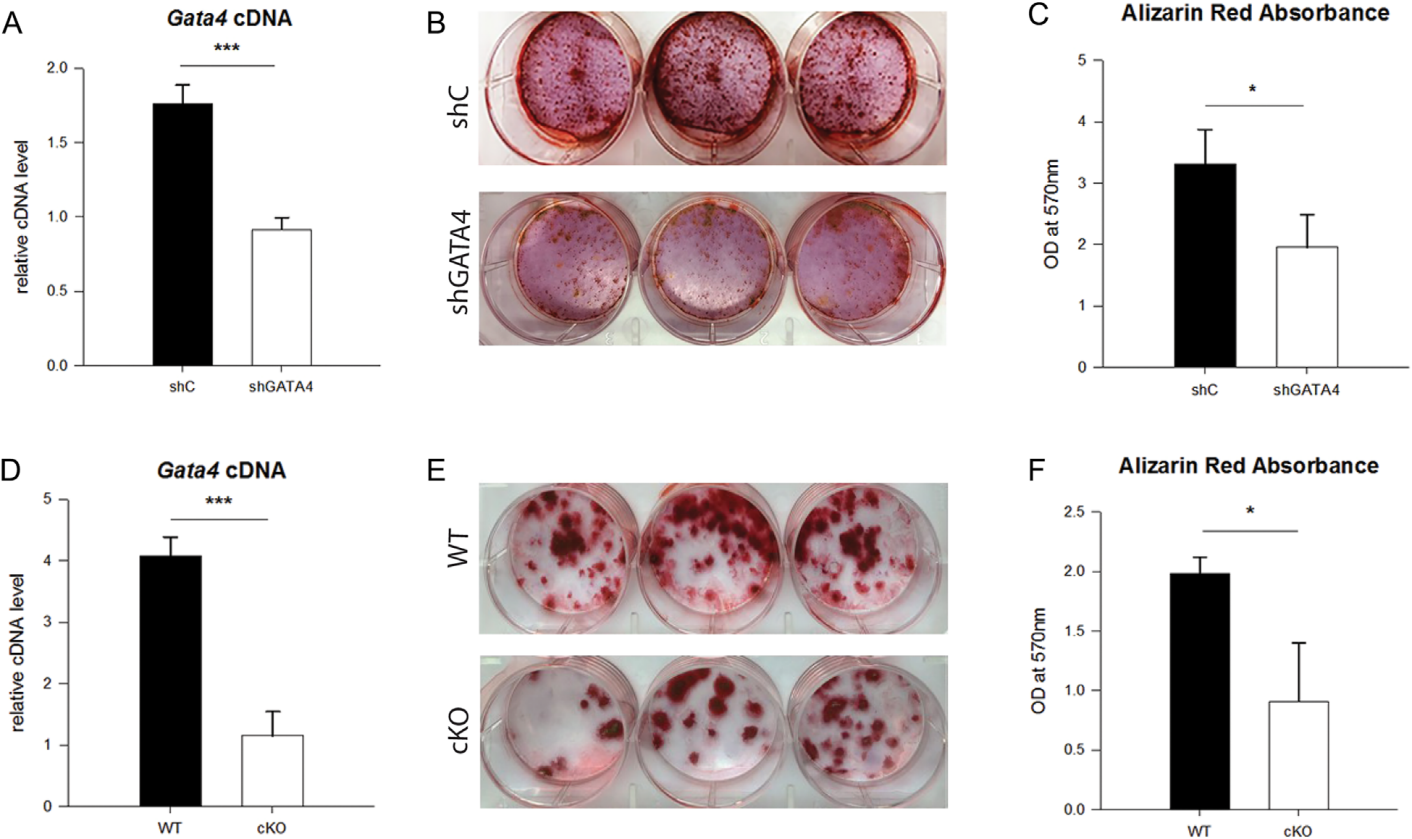
GATA4 regulates mineralization in vitro. (*A*) Calvarial osteoblasts were transduced with lentivirus expressing shC or shGATA4. RNA was obtained and qPCR was performed for *Gata4* and normalized to *actb* (β‐actin) mRNA. (*B*) Calvarial osteoblasts transduced with lentivirus directed toward shC or shGATA4 and were cultured in osteogenic media for 2 weeks. Cells were then fixed and stained using Alizarin Red. (*C*) Alizarin Red from *B* was eluted and the mineral content measured at an OB of 570 nm. Silencing was performed in three wells and the average OD is displayed. (*D*) Bone marrow from WT and cKO mice were cultured in osteogenic media for 2 weeks. RNA was obtained and qPCR was performed for *Gata4* and normalized to *Actb* (β‐actin) mRNA. (*E*) Cells were then fixed and stained using Alizarin Red. Three different mice were tested and a representative image from one mouse is presented in triplicate. (*F*) Alizarin Red from *E* was eluted and the mineral content measured at an OB of 570 nm. The average OD from all wells is displayed. Student's *t* test: *p < 0.05, ***p < 0.001 compared with wild type. OD = optical density.

To confirm that GATA4 plays a crucial role in osteoblast differentiation and/or mineralization, primary calvarial osteoblasts, following *Gata4* (or control) knockdown with lentiviral shRNA, were differentiated for 14 days, and the cDNA expression level of *Gata4* and Alizarin Red mineralization were quantified. *Gata4* mRNA was reduced twofold in shGata4 samples, confirming that *Gata4* was successfully knocked down in shGata4 samples compared with control silencing (shC) (Fig. 2 *A*). The primary calvarial cells transduced with shC showed efficient mineralization as visualized by Alizarin Red staining, but when *Gata4* was silenced, there was a significant reduction in Alizarin Red staining (Fig. 2 *B*). The mineral content, measured by eluting Alizarin Red, was significantly reduced in shGata4 silenced cells (Fig. 2 *C*). To further confirm that GATA4 affects mineralization, bone marrow stromal cells were isolated from WT and cKO femur and tibia and differentiated ex vivo for 14 days, and the *Gata4* expression level and mineralization were quantified. The *Gata4* expression was significantly reduced in cKO mice compared with WT mice (Fig. 2 *D*), along with reduced Alizarin Red staining and a significant reduction in mineral content (Fig. 2 *E*, *F*). This demonstrates that GATA4 affects mineralization both in primary calvarial osteoblasts, as well as in bone marrow‐derived osteoblasts from adult mice.

### GATA4 regulates RUNX2 in vitro

**Figure 3:**
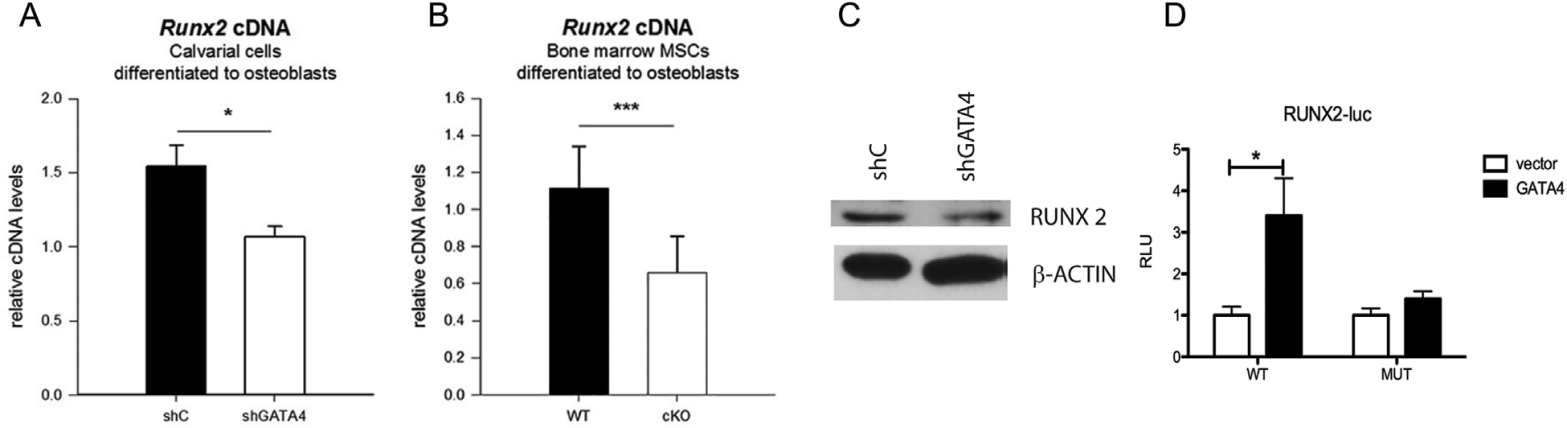
GATA4 regulates *Runx2* in both primary calvarial cells and in bone marrow MSCs. (*A*) Calvarial osteoblasts were transduced with lentivirus expressing shC or shGATA4. Cells were then differentiated for 2 weeks in osteogenic media and RNA was obtained. qPCR was performed for *Runx2* and normalized to *Actb* (β‐actin) mRNA. (*B*) Bone marrow was isolated from tibia and femurs of wild‐type and conditional GATA4 knockout mice. Cells were then differentiated for two weeks in osteogenic media and RNA was obtained. qPCR was performed for *Runx2* and normalized to *Actb* (β‐actin) mRNA. (*C*) Calvarial osteoblasts were transduced with lentivirus expressing either shC or shGATA4. Cells were then differentiated for 2 weeks in osteogenic media and protein was obtained and immunoblots for RUNX2 and β‐actin were performed. (*D*) The *Runx2*‐promoter luciferase construct was transfected into U2OS cells, along with pcDNA3‐GATA4, where indicated. The *Runx2*‐promoter mutant has two point mutations in the sequence “GATA.” Luciferase values were normalized to Renilla luciferase. *n* = 3, Student's *t* test: *p < 0.05, ***p < 0.001.

We had previously demonstrated that *Gata4* and *Runx2* are co‐expressed early in osteoblastogenesis.^6^ The in vivo data demonstrate that GATA4 regulates Runx2. Therefore, to test if GATA4 regulates *Runx2* in vitro, primary calvarial osteoblasts were silenced for *Gata4*, as described above. In a parallel experiment, bone marrow stromal cells from *Gata4* cKO and WT mice were differentiated to osteoblasts (Fig. 3 *A*, *B*). Knockdown of *Gata4*, as well as *Gata4* cKO mice, showed significant reductions in *Runx2* mRNA expression. We confirmed that absence of GATA4 has a functional consequence as knockdown of *Gata4* in the primary calvarial osteoblasts exhibits reduced RUNX2 protein levels (Fig. 3 *C*). Together, these data demonstrate a misregulation of *Runx2* in *Gata4*‐ablated cells.

*Runx2* is regulated by two different promoter regions: one specific for osteoblasts (P1) and the other promoter region specifically for both osteoblast and/or other mesenchymal tissues (P2).^17^ To determine if GATA4 regulates *Runx2* by binding to the *Runx2* promoter, a luciferase assay was performed, using the osteoblast‐specific P1 promoter.^17^ U2OS cells were transiently transfected with the *Runx2*‐promoter luciferase reporter plasmid, along with pcDNA3‐GATA4 or control pcDNA3 plasmid. GATA4 increased the luciferase expression by more than threefold. When the canonical GATA4 binding motif “GATA” was mutated to “GCCA,” GATA4 failed to induce luciferase activity (Fig. 3 *D*). These results suggest that *Runx2* transcriptional activity is activated by GATA4 both in vivo and in vitro.

### GATA4 regulates epigenetic modifications on the chromatin for *Runx2* expression

**Figure 4:**
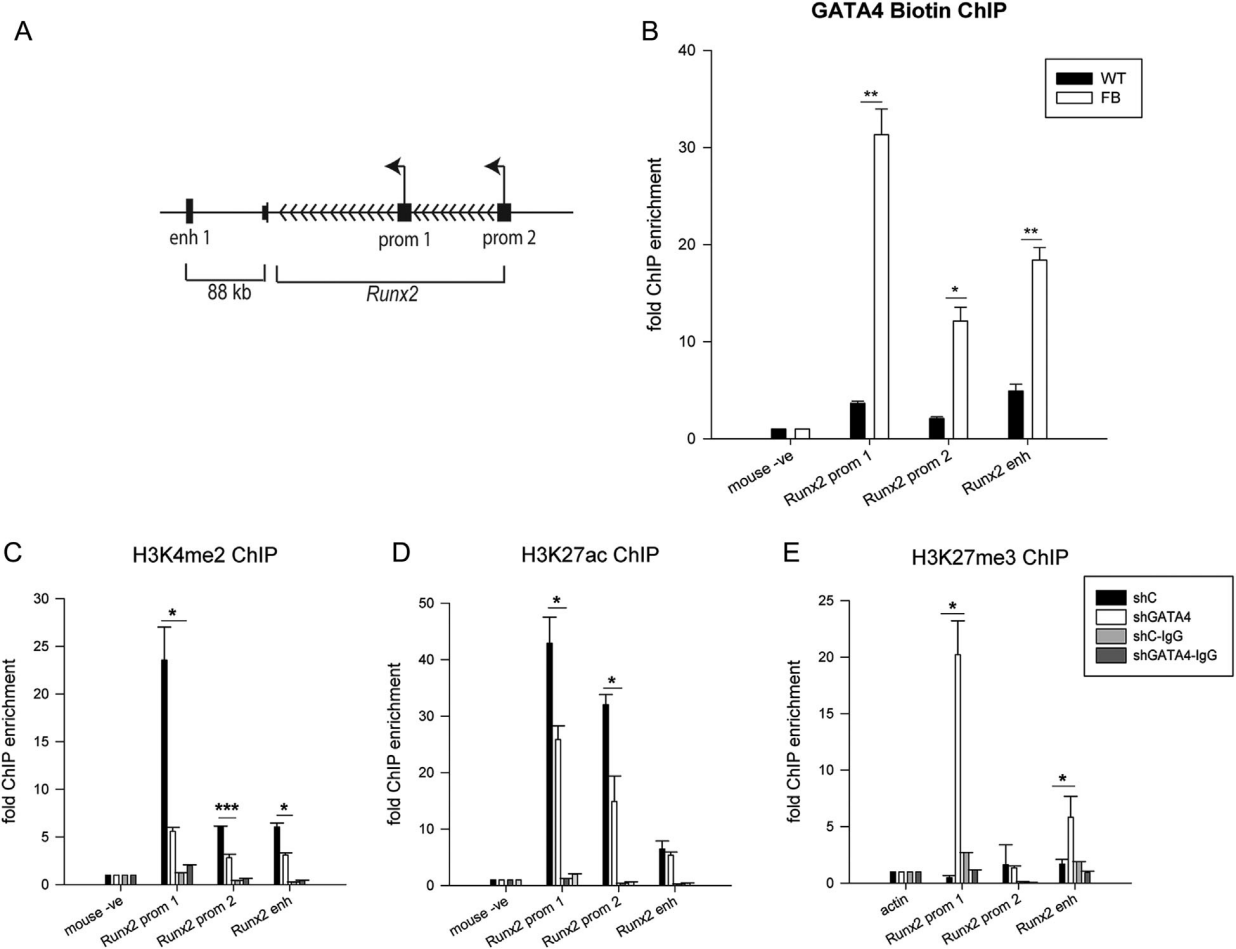
GATA4 regulates *Runx2* at two promoters and an enhancer. (*A*) Schematic diagram of *Runx2* genomic locus. The arrow indicates the direction of gene transcription. The GATA4 binding sites are *Runx2* promoter 1, promoter 2, and an enhancer site, which are represented by solid squares and triangle. (*B*) Streptavidin‐coated beads were used to immunoprecipitate either wild‐type or Flag‐biotin‐GATA4. qPCR was performed with primers to the indicated regions. Calvarial osteoblasts were transduced with either shC or shGATA4 lentivirus. ChIP was performed with antibodies to (*C*) H3K4me2, (*D*) H3K27ac (open chromatin), (*E*) H3K27me3 (closed chromatin), or IgG. qPCR was performed to the indicated regions. Each PCR was normalized to input and represented as fold enrichment over a negative genomic locus (H3K4me2 and H3K27ac: mo. −ve, and H3K27me3: actin). *n* = 3, Student's *t* test: *p < 0.05, ***p < 0.001 compared with shC.

Based on the above, we hypothesized that GATA4 directly regulates RUNX2 by directly binding to chromatin at promoter regions that are essential for regulating *Runx2* gene expression in normal osteoblast differentiation. To determine the precise binding loci in cells requires chromatin immunoprecipitation (ChIP) by GATA4 antibodies; unfortunately, commercially available antibodies do not perform well in ChIP. Therefore, a mouse with GATA4 tagged with the Flag epitope and a biotinylation site at the endogenous locus was created and has been successful for GATA4 ChIP.^18^, ^19^ Calvaria from these mice were obtained and ChIP was performed with streptavidin beads. Our schematic illustration defines both promoter regions and an enhancer region expected to be occupied by GATA4 (Fig. 4 *A*). We had identified an enhancer region for *Runx2* associated with open chromatin, marked by histone 3 lysine 4 dimethylation (H3K4me2) in human osteoblasts that we translated to the mouse genome (visualized in the UCSC Genome Browser). The isolated DNA was assayed for regions near the *Runx2* gene and to a negative control genomic region (Fig. 4 *B*). GATA4 was found to be occupied in all three regions of the *Runx2* loci, and the highest occupancy was found at promoter 1, which is specific for osteoblast differentiation, and less occupancy at promoter 2 and the enhancer region that is necessary for both osteoblasts and/or other mesenchymal tissues.

Mechanistically it is likely that GATA4 helps maintain the chromatin regions regulating *Runx2* expression to be “open” and/or epigenetically marked for transcription.^19^ Specific epigenetic marks that have been associated with GATA4 marking “open” chromatin near genes that are actively transcribed are histone 3 lysine 27 acetylation (H3K27ac), due to its association with the acetyltransferase p300,^19^ and H3K4me2. In contrast, repressed genes are marked by histone 3 lysine 27 trimethylation (H3K27me3). To determine if GATA4 affects these histone marks on either the *Runx2* proximal promoters and/or the enhancer region, we analyzed the histone marks at those sites in primary calvarial osteoblasts by ChIP in the presence and/or absence of GATA4 (Fig. 4 *C–E*). The two promoter sites and one enhancer site are positive for the open marks H3K4me2 (Fig. 4 *C*) and H3K27ac (Fig. 4 *D*), and negative for the closed mark H3K27me3 (Fig. 4 *E*) in normal calvarial osteoblasts. More importantly, when *Gata4* is silenced, both the H3K4me2 and H3K27ac open marks are significantly reduced; concurrently, the H3K27me3 mark is increased over baseline at promoter 1 and the enhancer region but not on the promoter 2 site.

U2OS osteosarcoma cells are a good model for osteoblasts,^20^, ^21^ and GATA4 biology can be largely reproduced in this cell line.^6^ Indeed, GATA4 can be localized by ChIP to the *Runx2* promoters and enhancer region, as in primary calvarial osteoblasts, and silencing of *Gata4* reduces *Runx2* expression (Supplemental Fig. S7*A*). Silencing of *Gata4* in U2OS cells also reduces the H3K4me2 and H3K27ac at the *Runx2* loci (Supplemental Fig.  7*C*, *D*).

**Figure 5:**
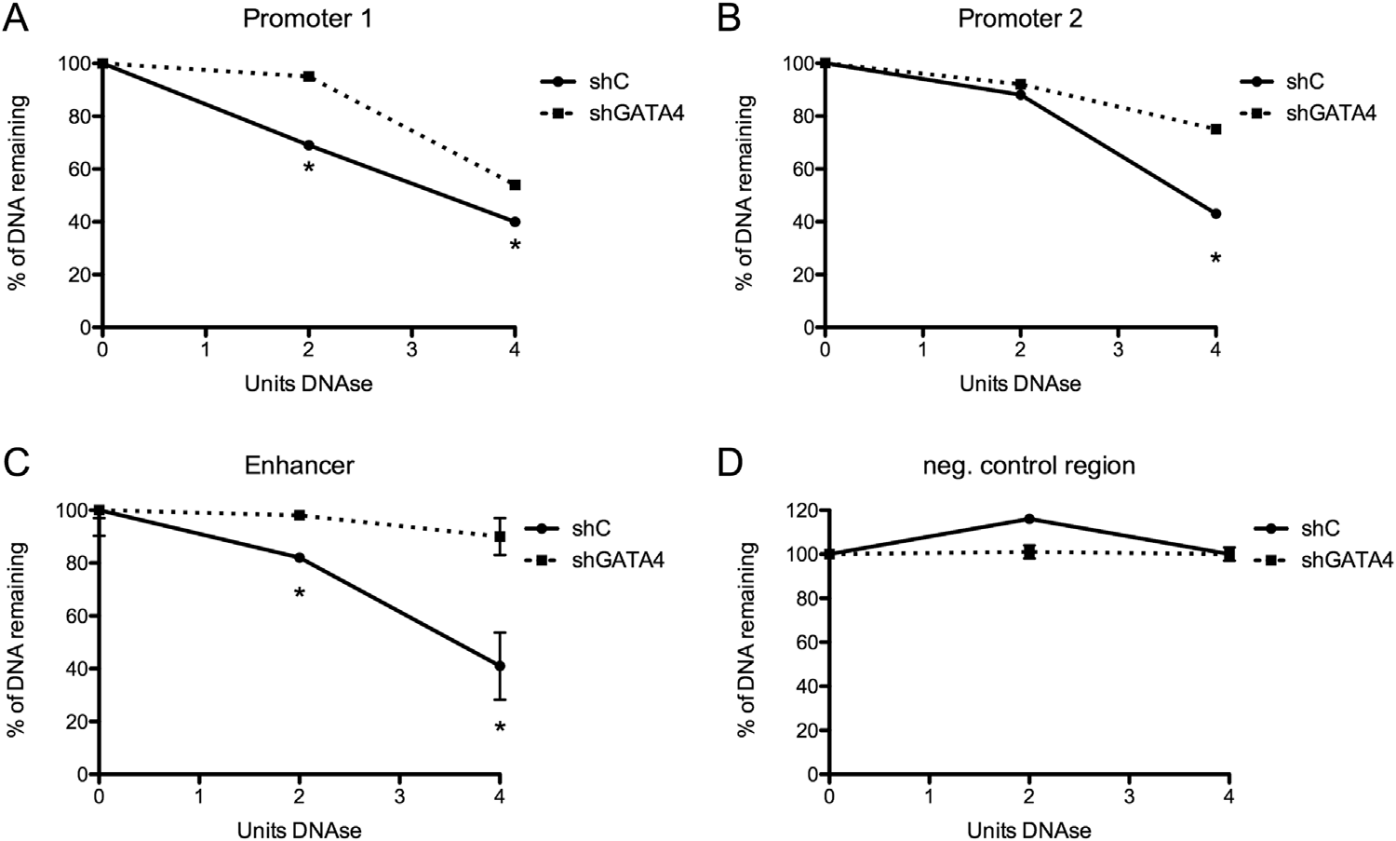
DNase assays demonstrate GATA4 is necessary for open chromatin at the *Runx2* locus. (*A–D*) *Gata4* was knocked down in primary calvarial osteoblast cells with lentivirus and then DNase assays were performed at the indicated *Runx2 cis* regulatory regions. *n* = 3, Student's *t* test: *p < 0.05.

Detection of chromatin remodeling can be determined by DNase sensitivity assays. Therefore, to further confirm if these *cis* regions have “open” or “closed” chromatin, DNase assays were performed in primary calvarial osteoblasts (Fig. 5) and U2OS cells (Supplemental Fig. S8). Promoter 1, promoter 2, and the enhancer have “open” chromatin and are accessible to DNase. However, when *Gata4* is knocked down, these regions become less accessible to DNase. Together, the DNase and ChIP experiments demonstrate that GATA4 maintains open chromatin near *Runx2* to regulate *Runx2* expression.

### GATA4 functions early in osteoblastogenesis to maintain “open” chromatin to induce RUNX2 expression

**Figure 6:**
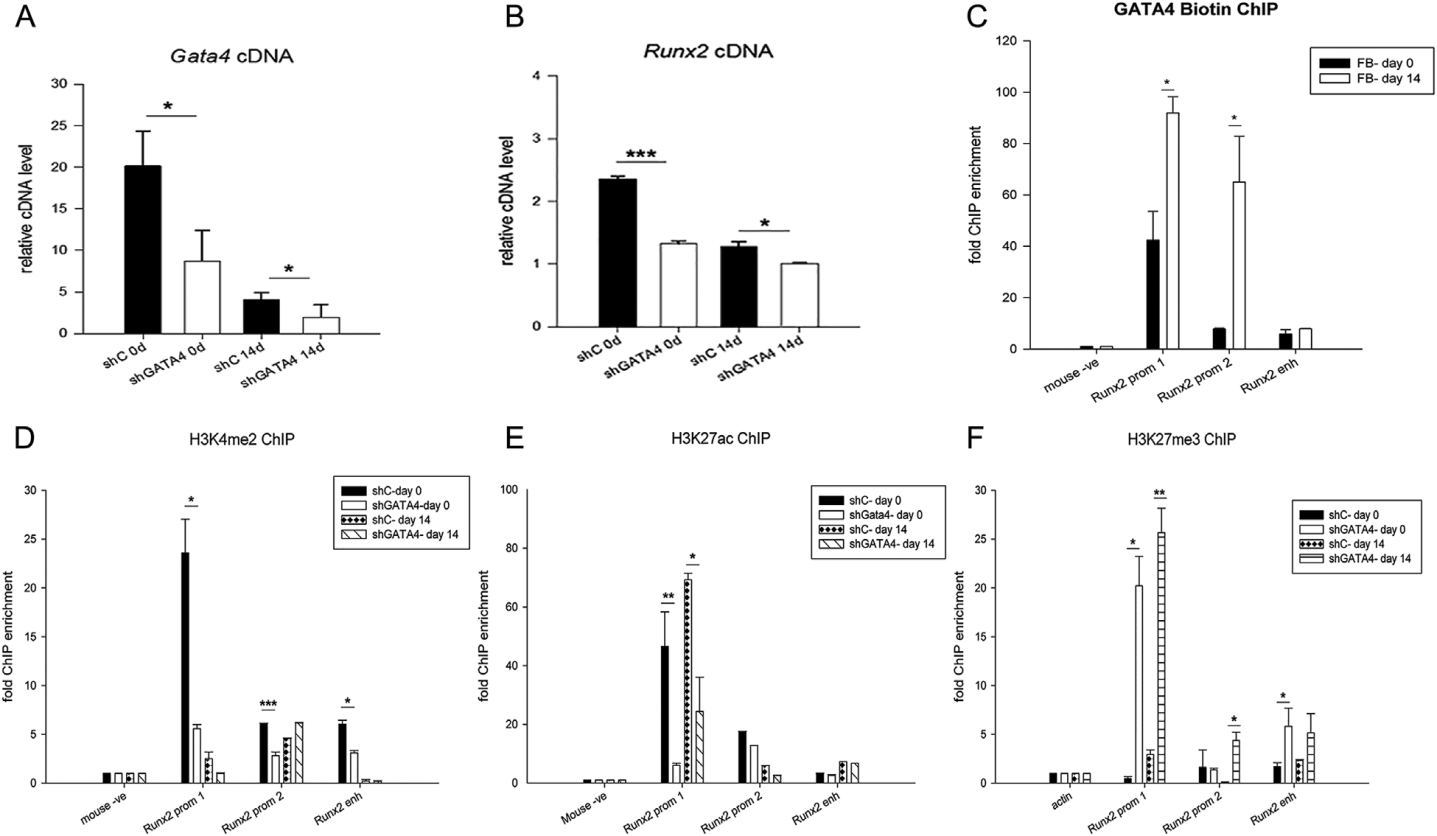
GATA4 regulates *Runx2* gene transcription early in differentiation. Calvarial osteoblasts were infected with lentivirus expressing shC or shGATA4. Cells were either collected 48 hours after infection (day 0) or differentiated for 2 weeks in osteogenic media. RNA was obtained from undifferentiated and differentiated cells and qPCR was performed for (*A*) *Gata4* and (*B*) *Runx2* and normalized to *Actb* (β‐actin) mRNA. (*C*) ChIP was performed with streptavidin beads on undifferentiated (day 0) or differentiated (day 14) osteoblasts from GATA4‐Flag‐Biotin mice. ChIP was performed with an antibody to (*D*) H3K4me2 (open chromatin), (*E*) H3K27ac, or (*F*) H3k27me3 and qPCR was performed to detect GATA4 binding sites at *Runx2* promoters and enhancer region. Each qPCR was normalized to input and represented as fold enrichment over a negative genomic locus (mo. −ve) or the actin (*Actb*) promoter. *n* = 3, Student's *t* test: *p < 0.05, **p < 0.01, ***p < 0.001.

*Gata4* and *Runx2* expression decrease late in osteoblastogenesis (Fig. 6 *A*, *B*). At day 14 of differentiation, the cells are mineralizing the matrix, as evidenced by Alizarin Red staining (Fig. 2). At this point, *Gata4* mRNA is more than 10‐fold lower than at day 0. Concurrently, *Runx2* mRNA is also decreased at day 14. At these same time points, GATA4 remains localized to the *Runx2* promoters and enhancer (Fig. 6 *C*). In undifferentiated cells, H3K4me2 at the osteoblast‐specific promoter 1 is 13‐fold higher than at day 14 (Fig. 6 *D*). *Gata4* knockdown no longer has a significant effect on H3K4me2 at day 14 at any of the three *Runx2* regulatory regions. Similarly, H3K27ac at promoter 1 is decreased by *Gata4* silencing at day 0 but not at day 14 (Fig. 6 *E*). H3K27me3 is increased by *Gata4* silencing at promoter 1 and the enhancer at day 0 and at promoter 1 and the enhancer at day 14 (Fig. 6 *F*). Thus, GATA4 functions to directly control *Runx2* expression in a temporal and spatial manner.

## Discussion

*Gata4* is expressed in mesenchymal stem cells and in osteoblastogenesis.^5^ It regulates many osteoblast genes, such as alkaline phosphatase, estrogen receptor alpha, and Fas Ligand.^5^, ^6^ These experiments, herein, demonstrate that GATA4 also directly regulates *Runx2* expression, by opening the chromatin region and having a direct effect on bone properties in vivo.

*Gata4* knockout in mice is embryonic lethal.^3^, ^4^ Conditional *Gata4* knockout in mice (cKO) with Cre expression driven by the 2.3‐kb promoter of *Col1a1* is partially embryonic lethal,^5^ with a small percentage of mice surviving to birth and adulthood. It is rare for a gene knocked out specifically in bone to be embryonic lethal. Surprisingly, the majority of the *Gata4* cKO mice did not survive to adulthood, possibly because of cranial or spinal defects. The neonatal and the adult *Gata4* cKO mice had lower BV/TV, decreased trabecular number, and increased trabecular spacing. We further describe that GATA4 regulates *Runx2* expression via binding directly to three loci near *Runx2*.

Our results determined that GATA4 regulates *Runx2* expression in cKO mice with the Col1A1 2.3‐kb promoter‐Cre expression, causing a conundrum, as it is believed that Col1A1 expression begins at E14.5 and *Runx2* expression peaks at E12.5. However, *Runx2* is expressed throughout osteoblastogenesis, as is *Gata4*. It is possible that GATA4 is necessary to maintain open chromatin near *Runx2*, instead of initializing chromatin opening. The Col1A1 2.3‐kb‐cre;ROSA26 (R26R LacZ) mouse demonstrates a high level of bone‐specific expression of Cre recombinase;^22^ however, the cKO mice described here also have chondrocyte defects, possibly because of expression of the Col1A1 2.3‐kb‐Cre in chondrocytes.^23^ It is also possible that GATA4 has non‐cell autonomous effects, effecting early osteoblastogenesis and chondrogenesis, and requiring the generation of linage tracing models, in future work, to address this possibility.

GATA4 has been shown to direct tissue‐specific gene expression, particularly in the heart and liver.^19^, ^24^ For example, GATA4 is one of the first proteins to bind to the albumin promoter in the embryo to drive liver specification.^25^ GATA4, along with HNF3/FoxA proteins, allows for stable nucleosome binding and the formation of the enhancer complex.^26^ GATA4 interacts with p300 to induce H3K27 acetylation in enhancer regions, opening the chromatin.^19^ Our ex vivo and in vivo modeling, in the absence or presence of *Gata4* expression, allowed, for the first time, a precise mapping of the chromatin epigenetic remodeling regulating *Runx2* mRNA expression. These data provide very strong evidence, mechanistically, how and when *Runx2* can either influence both osteoblast differentiation alone and/or concurrently regulate other mesenchymal tissue (ie, chondrocyte differentiation) in vivo. At very specific osteoblast differentiation timing, in adult mice, we identified differential occupancy of *Gata4* on both the promoters and an enhancer. In support of this notion, we provide in vivo analysis of RUNX2 protein alteration in the trabecular bone at the growth plate and/or near the hypertrophic chondrocytes, which happens only during endochondral ossification. The direct role of GATA4 and/or RUNX2 on chondrocyte differentiation will require further in vivo and in vitro experiments. Here, we propose that GATA4 helps drive osteoblast‐specific gene expression by regulating open chromatin. Whether H3K27ac is dependent on p300 at the RUNX2 loci will also need to be experimentally confirmed in future work.

GATA4's regulation of osteoblast differentiation is multifactorial. With estrogen receptor alpha, GATA4 regulates FasL, FoxC1, and alkaline phosphatase.^6^, ^27^ GATA4 has also been shown to regulate osteogenic differentiation by directly regulating *Gnai3* and bone sialoprotein.^28^, ^29^ Genomewide localization (ChIP‐sequencing) of GATA4 at the DNA has been performed in the liver and in the heart.^19^, ^30^ Only about 5% of the more than 16,000 binding sites for GATA4 overlapped between the two tissues. In the liver, GATA4 binds near genes involved in metabolism of lipids and lipoproteins, glycolysis, cytochrome P450 metabolism, and more. In the heart, GATA4 regulates tissue‐specific pathways, including cardiac muscle, chamber, and ventricle development and heart growth.^19^ GATA4 only binds to the proximal promoter of genes about 17% of the time. Based on these data sets, predicting the binding sites for GATA4 in the osteoblast probably cannot be predicted from these other data sets or from expression data, and ChIP sequencing of GATA4 in osteoblasts would reveal tissue‐specific regulatory regions.

RUNX2 activity is regulated by transcription, protein‐protein interactions,^31^ posttranslational modifications (including phosphorylation,^32^ acetylation,^33^ and sumoylation^34^), and protein degradation.^35^ BMPs have been shown to upregulate *Runx2* expression.^36^, ^37^ In addition, TGFβ inducible early gene‐1 (TIEG1)^38^ has been shown to bind to the *Runx2* promoter to drive *Runx2* expression. We have previously demonstrated that GATA4 regulates BMP and TGFβ signaling pathway components in osteoblasts.^5^ Thus, GATA4 regulates *Runx2* expression directly at the *Runx2* gene and indirectly via regulating the BMP and TGFβ pathways.

## Disclosures

All authors state that they have no conflicts of interest.

## Supporting information

Supporting Data S1.
