# Supplementary material for: GATA4 Directly Regulates Runx2 Expression and Osteoblast Differentiation
Source: JBMR Plus. 2018 Jan 3;2(2):81–91. doi: 10.1002/jbm4.10027 (PMC6053063; doi:10.1002/jbm4.10027)
Supplement: Supplementary file 1 — Supporting Data S1. [file JBM4-2-81-s001.pdf]

SUPPLEMENTAL Figure 1

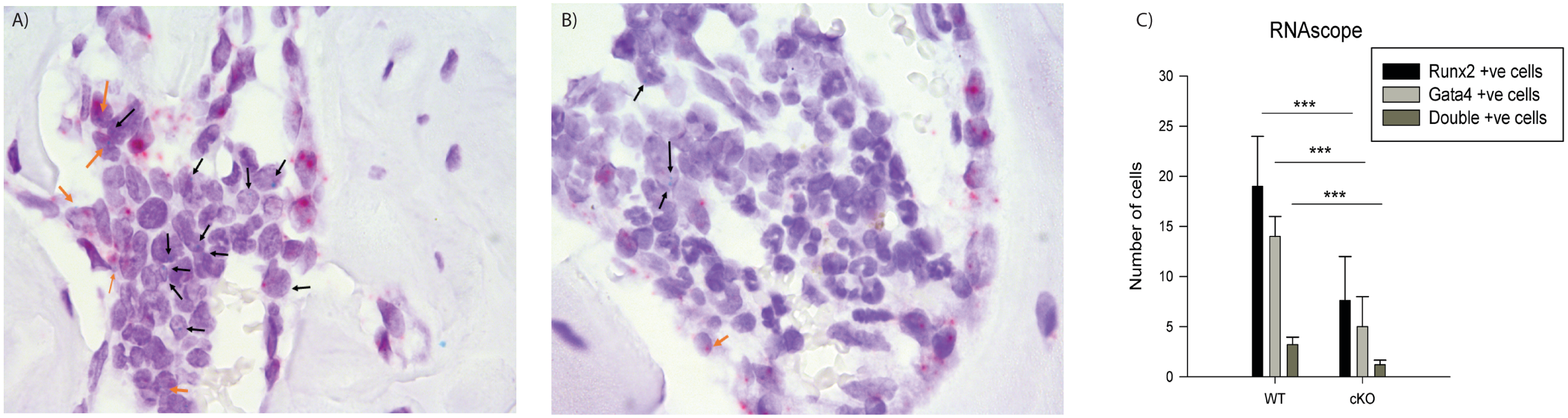

WT

cKO

SUPPLEMENTAL Figure 2

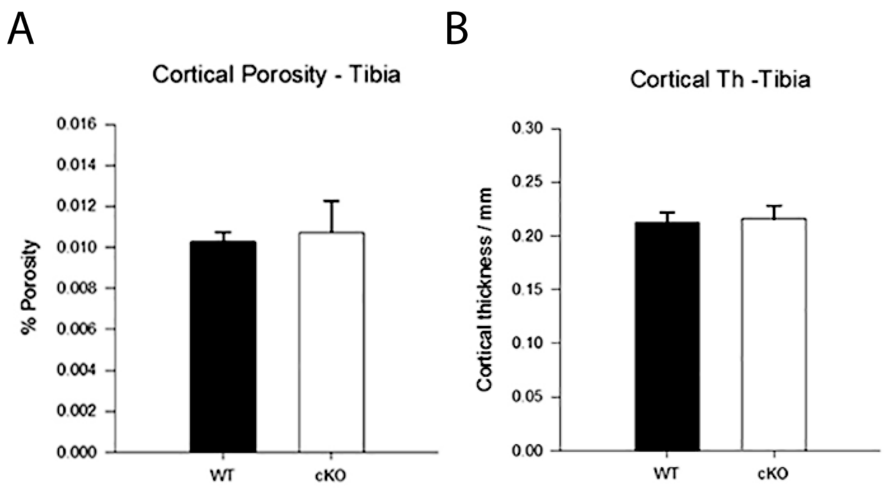

Supplemental FIGURE 3

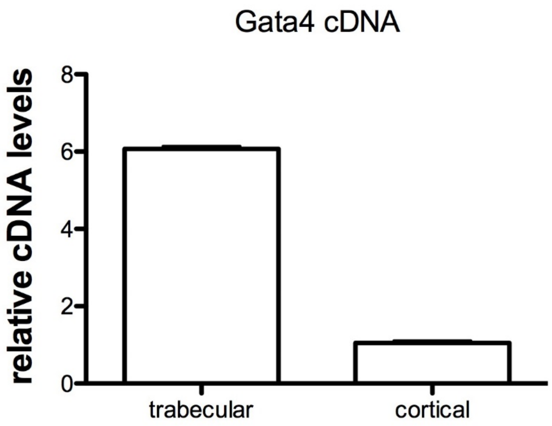

SUPPLEMENTAL Figure 4

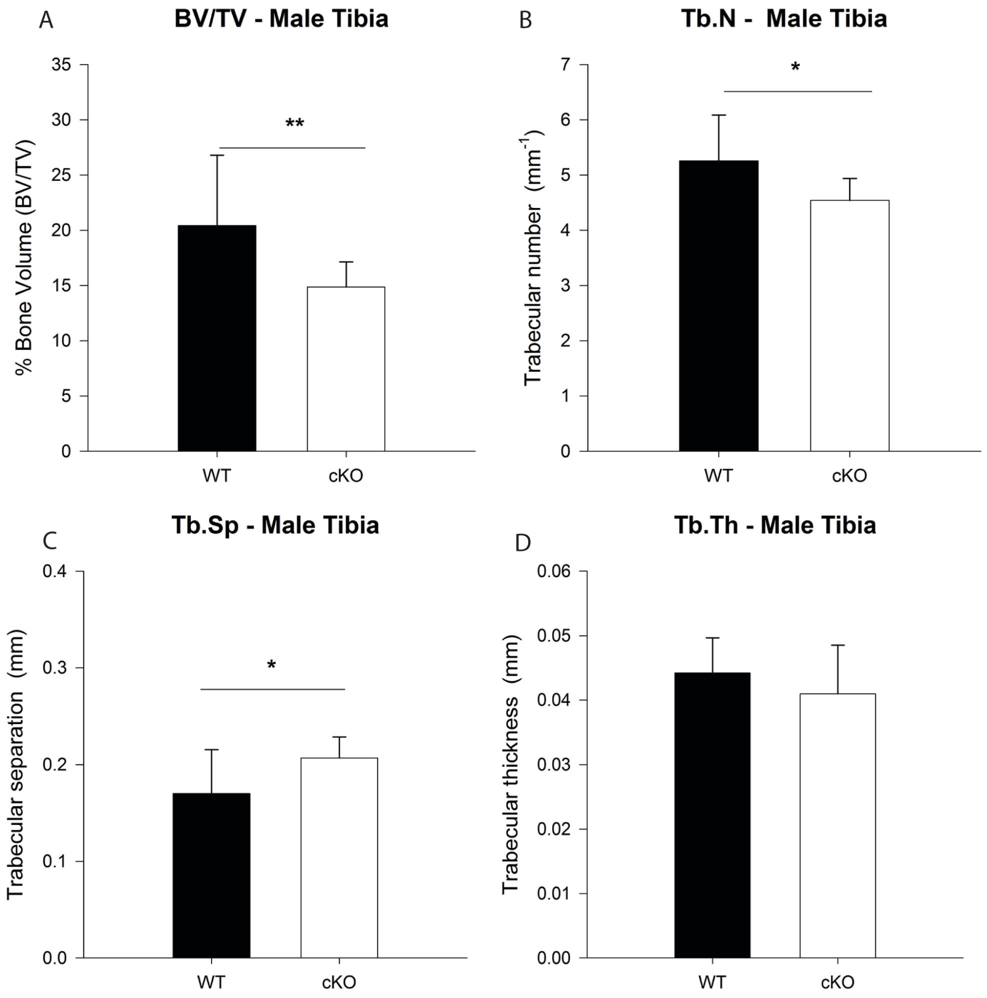

Supplemental FIGURE 5

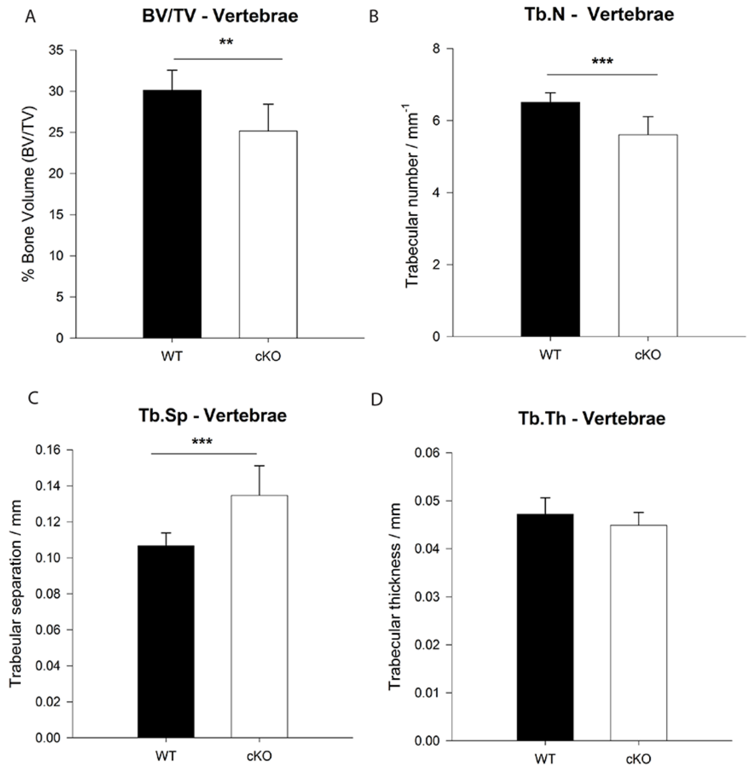

Supplemental FIGURE 6

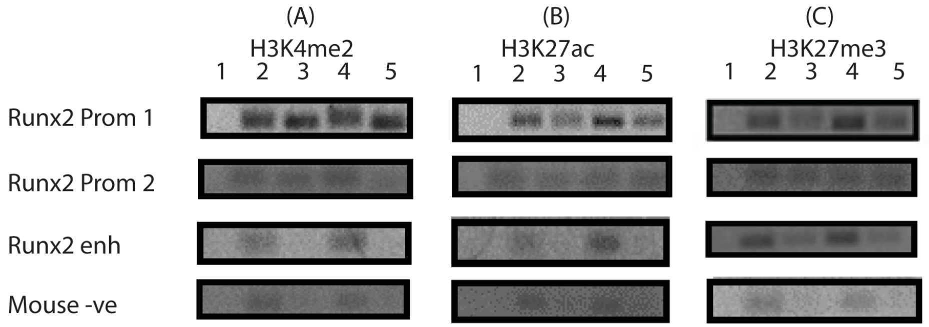

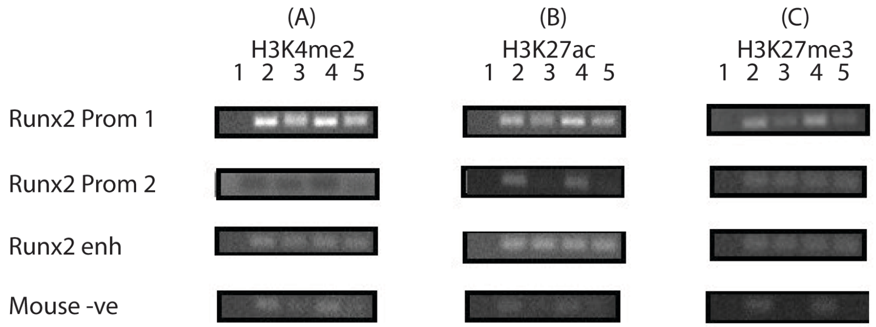

Supplemental FIGURE 7

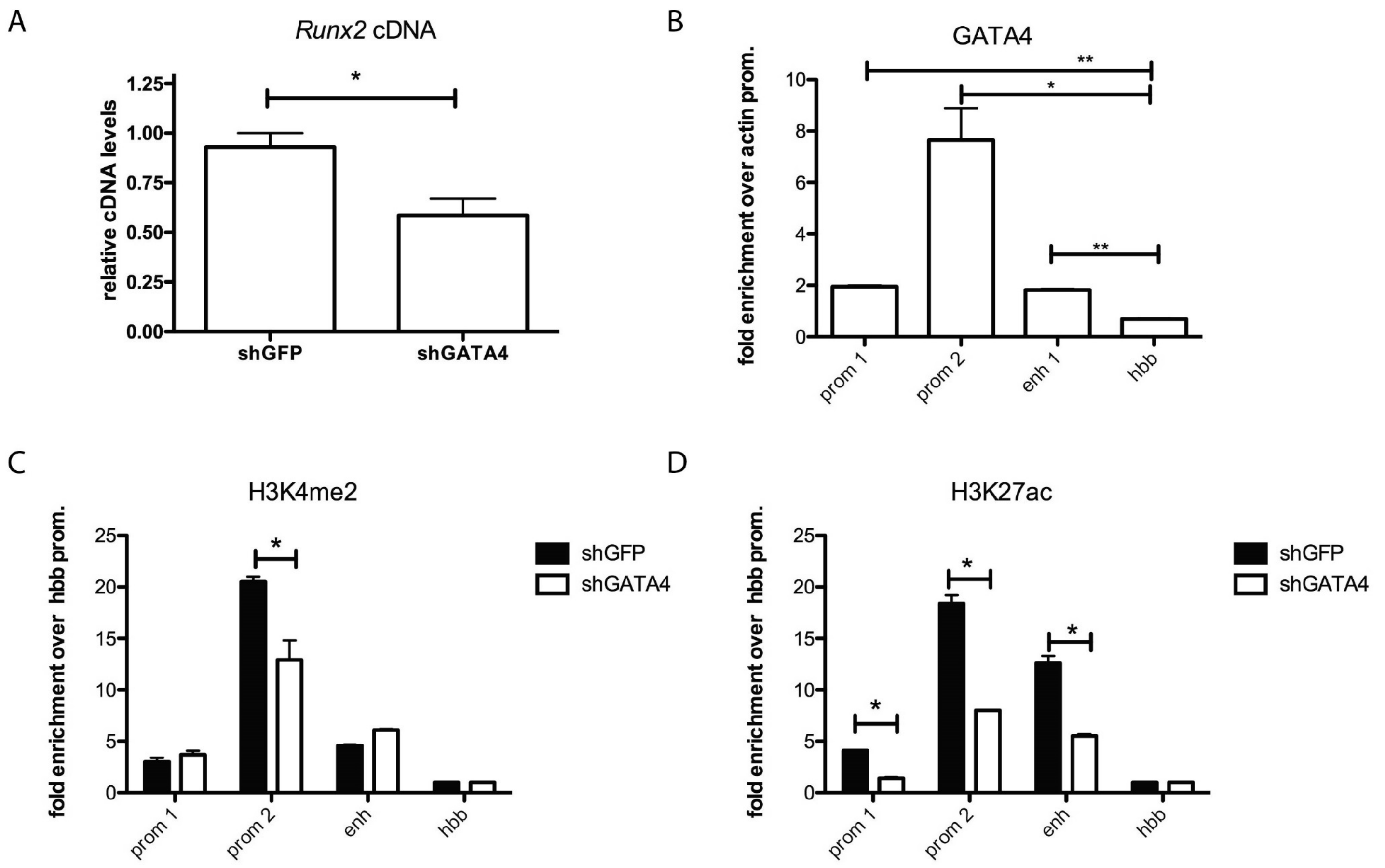

Supplemental FIGURE 8

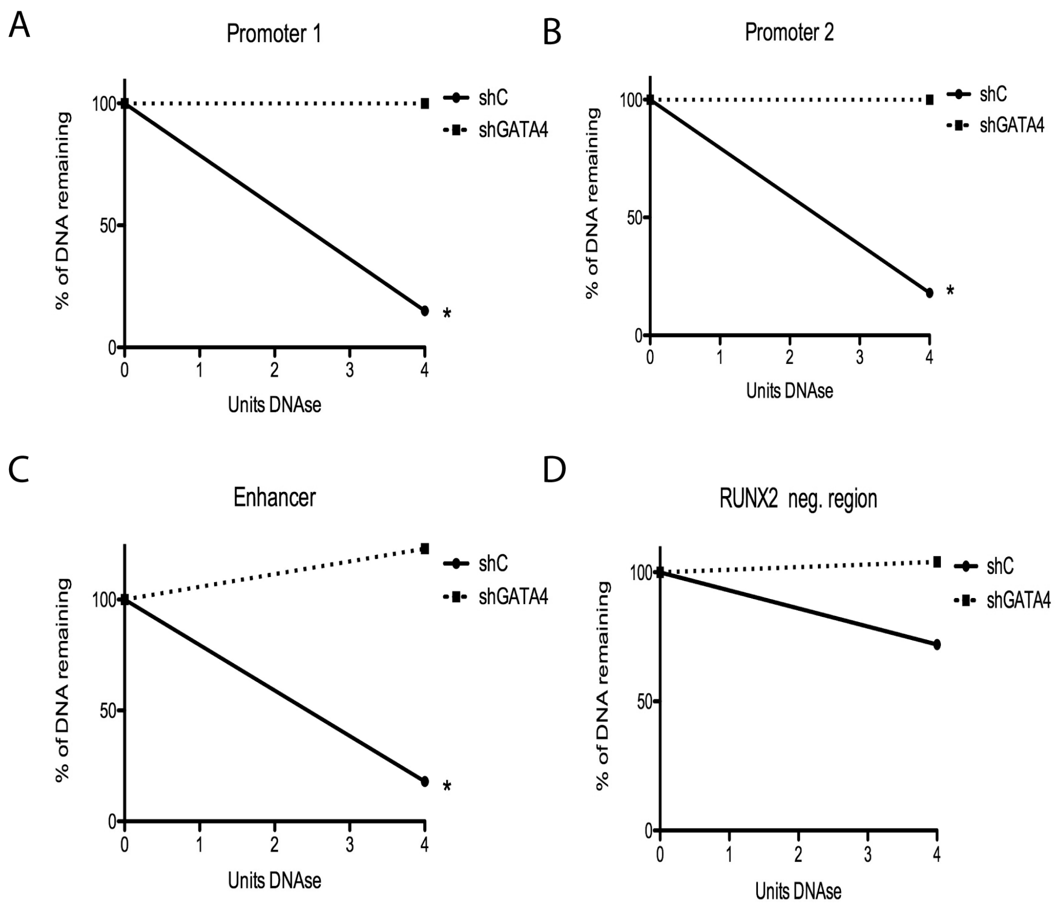

Supplemental Table. 1. Micro CT analysis of femurs from 14-week-old male and female wildtype or cKO mice

	Male			Female	
Section	Wildtype	cKO		Wildtype	cKO
BV/TV (%)	21.1 ± 4.1	17.3 ± 2.4*		18.8 ± 5.1	14.5 ± 2.1**
Tb. Th (mm)	0.044 ± 0.004	0.040 ± 0.007		0.041 ± 0.004	0.040 ± 0.002**
Tb. N (1/mm)	5.43 ± 0.57	4.95 ± 0.42		4.75 ± 0.84	4.71 ± 0.45*
Tb. Sp (mm)	0.16 ± 0.03	0.18 ± 0.01		0.18 ± 0.04	0.18 ± 0.02

Supplemental Table 2

**Primers for cDNA**		species
Actin (ACTB)	agccatgtacgtagccatcc	mouse
	ctctcagctgtggtggtgaa	
Runx2	cacggtgactcccgttactt	mouse
	atacgtgtgacccagtgcaa	
Actin (ACTB)	agcactgtgttggcgtacag	human
	aaactggaacggtgaaggtg	
Runx2	tttgcactgggtcatgtgtt	human
	tggctgcattgaaaagactg	
Gata4	tccctcttccctcctcaaat	human
	tcagcgtgtaaaggcatctg	
**Taqman**	**ThermoScientific Assay #**	
Actin	Mm00607939_s1	mouse
Gata4	Mm00484689_m1	mouse
**Primers used for ChIP**		
Actin promoter (ACTB)	GATGCTGACCCTCATCCACT	mouse
	ATGAAGAGTTTTGGCGATGG	
Runx2 prom 1	ACCTTCTGAATGCCAGGAAGGCCT	mouse
	TGGGACTGCCTACCACTGTGG	
Runx2 prom 2	TCCTGGACCAGAGCCACGTT	mouse
	GCAACTCCCTGGATGCCCTGG	
Runx2 enh	CCCCCAAAGTGGGGAGTGGC	mouse
	TGCTTGCTGGGAAAGGGGGA	
Mouse -ve	GTGGCTGCTCTTCGCTGCCA	mouse
	TGCTGCCACCTGGCCTTTGG	
Actin promoter (ACTB)	AGTGCCCAAGAGATGTCCAC	human
	AGAGCGAGAGCGAGATTGAG	
Runx2 prom 1	TGAGCGGGGAGTAGAAAGGA	human
	ACAAATGCTGAAGGAGCCCA	
Runx2 prom 2	CGAGAGACAGGGGAGCTTTG	human
	GCTAGGCTCCTTCCTTTCCC	
Runx2 enh	CGTGGGTCTTTTAGACAGACAA	human
	TTACGTTGATGTGATGAGGGCA	
HBB	TGGTATGGGGCCAAGAGATA	human
	TAGATGGCTCTGCCCTGACT	
Runx2 neg region	ATTTAGCCCCCGAAAACTTG	human
	GGTCCCACAGAGCAAGGATA	
